# Coupled chemo(enzymatic) reactions in continuous flow

**DOI:** 10.3762/bjoc.7.169

**Published:** 2011-10-24

**Authors:** Ruslan Yuryev, Simon Strompen, Andreas Liese

**Affiliations:** 1 Institute of Technical Biocatalysis, Hamburg University of Technology, Denickestr. 15, 21073, Hamburg, Germany

**Keywords:** biocatalysis, chemo-enzymatic reaction sequences, continuous flow, coupled reactions, reaction cascades

## Abstract

This review highlights the state of the art in the field of coupled chemo(enzymatic) reactions in continuous flow. Three different approaches to such reaction systems are presented herein and discussed in view of their advantages and disadvantages as well as trends for their future development.

## Introduction

**Figure 1:**
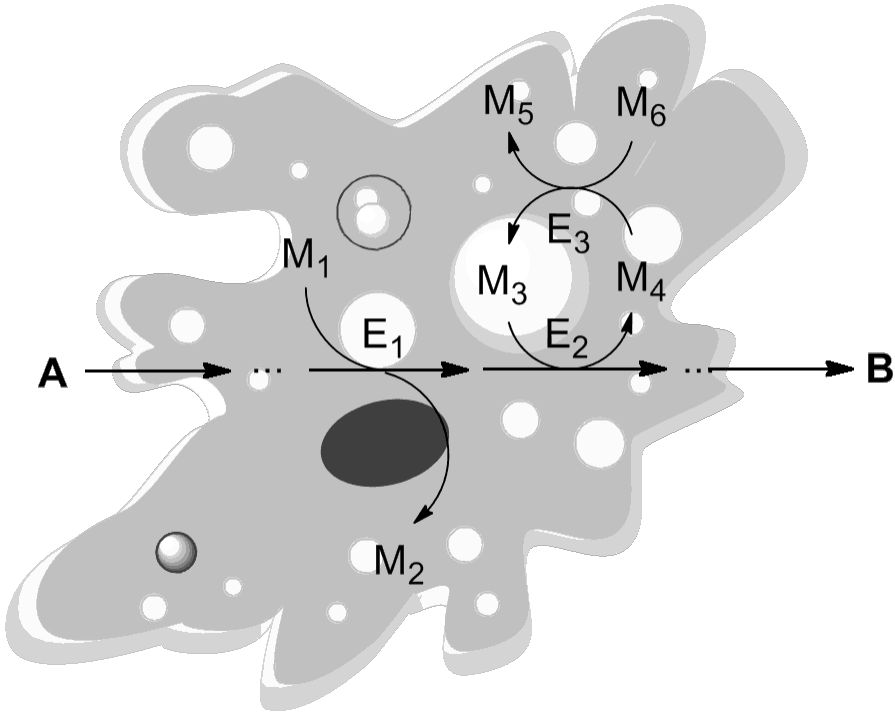
Metabolic pathways in a living cell as an example of efficient coupled-reaction processes. A: Substrate; B: Product; E_1–3_: Enzymes; M_1–6_: Metabolites.

For a long time, the living cell has been considered to be a perfect chemical factory, whose organizational principles can inspire every organic chemist and chemical engineer. The effectiveness, with which nutrients are converted into complex chemical building blocks required for the cell metabolism, is still a distant goal for any man-made chemical factory. Metabolic pathways, consisting of enzymatic sequential and coupled reactions (Figure 1), lie at the core of any living system and have been optimized by evolution over billions of years to create the phenomenon of life as we know it.

**Figure 2:**
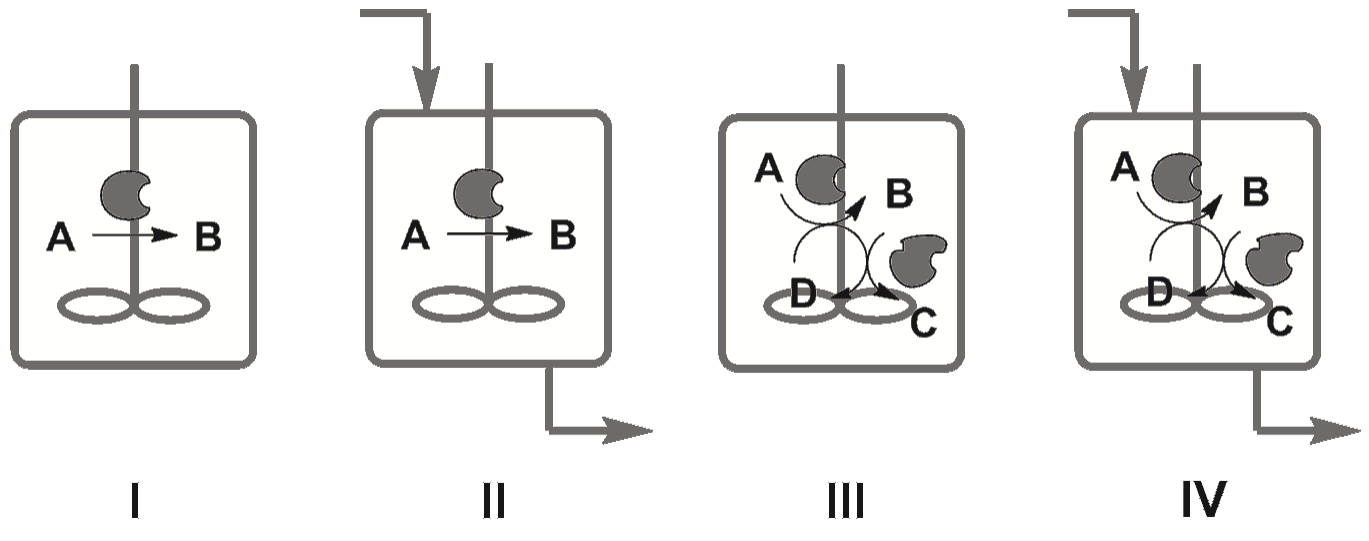
Four generations of biotransformations. I: Single-reaction processes; II: Single-reaction processes in continuous flow; III: Coupled-reaction processes; IV: Coupled-reaction processes in continuous flow.

In the field of applied biocatalysis, chemists are constantly trying to recognize the principles responsible for the efficiency of cell metabolism and to exploit them in organic synthesis [1–3]. There are three biological principles whose implementation may be regarded as important milestones in this field and which can be used for the classification of existing biotransformations. One of these principles is that a single reaction step of a given metabolic pathway proceeds in a very specific manner due to the intrinsically high chemo-, regio- and stereoselectivity of the enzyme catalyzing this step. This principle is the soul of applied biocatalysis and has already been widely exploited in the chemical industry for decades in the production of chemicals by enzymatic processes [4–6]. Biotransformations solely based on this principle, i.e., “single-reaction–single-enzyme” systems, may be classified as first-generation enzymatic processes (Figure 2, I), which historically were the first to be applied in the chemical industry and till now remain the most abundant among industrial biotransformations.

The second biological principle states that cell metabolism is a continuous process. Every metabolically active living cell is an open system that requires a constant flux of nutrients in order to stay alive. The numerous continuous chemocatalytic processes implemented on laboratory and on industrial scales prove that this principle can be effectively transferred to technical systems as well [7–11]. Biotransformations, which combine both of the biological principles mentioned, may be regarded as “single-reaction–single-enzyme continuous-flow” systems and classified as second-generation enzymatic processes (Figure 2, II). Such biotransformations naturally evolve from the corresponding first-generation “single-reaction” batch processes and are often economically more attractive than their ancestors due to the higher productivity that they afford. Therefore, it is not surprising that the second-generation biotransformations have received much attention in recent years [12].

The third biological principle says that cell metabolism is a complex network of reactions coupled through common substrates and products: Multistep syntheses of metabolites are conducted in sequential reactions catalyzed by spatially aligned enzymatic complexes, while coupled parallel reactions are used to regenerate costly cofactors or to enable thermodynamically unfavorable steps. The practical realization of this principle is a dream for every organic chemist who wishes to perform a multistep organic synthesis of a desired compound in one pot, without isolation of the intermediates. And this dream has recently become reality: In the last few decades the concept of reaction cascades has become increasingly popular and has been proven to be a viable synthetic route to many classes of organic compounds [13–15]. Biotransformations consisting of coupled sequential and/or parallel reactions catalyzed by one or several enzymes may be regarded as third-generation enzymatic processes (Figure 2, III). The interest in such systems is prompted by their obvious economical potential: In situ regeneration of expensive cofactors and reduction of downstream processing steps decreases production costs and generation of waste [16–17]. Therefore such processes are generally expected to have a higher “green index” [18] and E-factor (kilograms of total waste produced per kilogram of product) [19]. One may also consider so-called chemo-enzymatic reaction sequences, that is multistep-reaction systems in which chemical and biocatalytic reaction steps are coupled, as third-generation biotransformations. These hybrid systems, in which the strengths of both chemical and biological approaches are combined, have proven to be powerful tools for organic synthesis and thus have nowadays become a hot topic in applied biocatalysis [20]. From a formal point of view, reactions such as the lipase-catalyzed hydrolysis of triglycerides to glycerol and fatty acids, or amylase-catalyzed hydrolysis of amylose to glucose should also be classified as multistep-reaction processes, because they proceed stepwise via a sequence of intermediates. However, such transformations differ from the third-generation multistep-reaction enzymatic processes in the sense that every reaction step is formally the same, i.e., it is catalyzed by the same enzyme under the same reaction conditions and the intermediates are of the same nature. Therefore, reaction cascades catalyzed by a single enzyme belong instead to the first-generation processes.

When following the logic of the chosen classification of enzymatic processes, the final or fourth-generation biotransformations would be defined as coupled chemo(enzymatic) reactions in continuous-flow systems based on all three of biological principles mentioned (Figure 2, IV). From a technical point of view, biotransformations of the last generation are the most sophisticated ones most closely resembling the metabolic activity of a living cell. At the same time, however, due to their complexity, these biotransformations are not as widespread as the enzymatic processes of earlier generations and their development is still in an early stage. Nevertheless, they can already be considered as a complementary technology to multistep continuous-flow organic synthesis: A chemical technology that has recently emerged on the frontier of organic chemistry and chemical engineering [8,21]. In this work we present achievements and challenges of the fourth-generation biotransformations, and highlight existing trends in this field, which in the foreseeable future may lead to the next-generation enzymatic processes.

## Review

### 1. Single-reactor processes in vitro

**Scheme 1:**
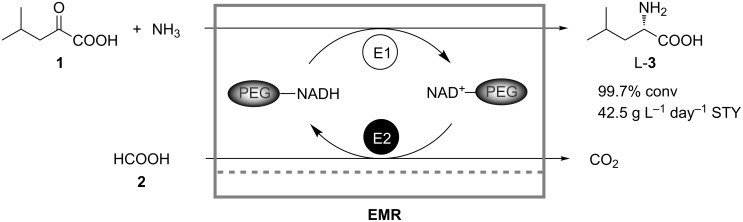
Production of L-leucine (**3**) in a continuously operating enzyme membrane reactor (EMR). E1: L-Leucine dehydrogenase; E2: Formate dehydrogenase [22].

Certainly, the most elegant and technically the most attractive way to perform coupled (chemo)enzymatic reactions is to bring all reactants and catalysts in contact in one vessel filled with one reaction medium. In this case the requirements with respect to equipment are reduced to a minimum. The single-reactor or so-called “in-pot” [17] coupled-reaction processes in continuous flow were established as early as 1981, most probably for the first time by Wichmann and Wandrey for the continuous production of L-leucine (**3**) in an ultrafiltration membrane reactor using a coupled parallel enzymatic system [22]. Here, L-leucine dehydrogenase was used for the reductive amination of α-ketoisocaproate (**1**) to the amino acid **3**. The required cofactor NADH was regenerated in a coupled parallel reaction by oxidation of formate (**2**) catalyzed by formate dehydrogenase (Scheme 1). Retention of the cofactor was achieved by its covalent binding to polyethylene glycol (PEG) with a molecular weight of 10 kDa. The reactor was operated continuously for 48 days under sterile conditions. A maximal conversion of 99.7% and a space-time yield (STY) of 42.5 g L^−1^ day^−1^ were reached.

**Scheme 2:**
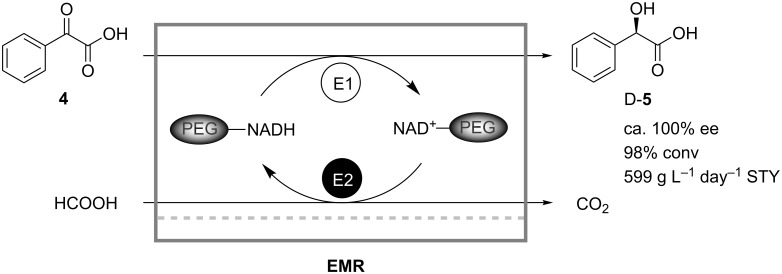
Production of D-mandelic acid (**5**) in a continuously operating enzyme membrane reactor. E1: D-(−)-Mandelic acid dehydrogenase; E2: Formate dehydrogenase [23].

Hummel and coworkers similarly described the stereoselective conversion of benzoyl formate (**4**) to D-mandelic acid (**5**) by a D-(−)-mandelic acid dehydrogenase from *Lactobacillus curvatus* in a sterilized enzyme membrane reactor, which was operated continuously (Scheme 2) [23]. Again, formate dehydrogenase was used for cofactor regeneration, and immobilization of NADH on PEG prevented loss of the cofactor in the continuously operated reactor. Space-time yields of 700 g L^−1^ day^−1^ were achieved with the optimized reactor at 95% conversion. Deactivation of enzyme and PEG–NADH led to a decrease of conversion to 90%. However, by adjustment of the residence time a continuous operation for 20 days was demonstrated.

**Scheme 3:**
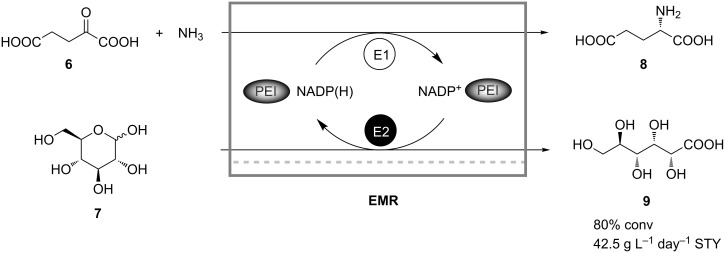
Simultaneous synthesis of gluconic acid (**9**) and glutamic acid (**8**) in a continuously operated membrane reactor. E1: Glutamate dehydrogenase; E2: Glucose oxidase [24].

Covalent grafting of cofactors on soluble polymers for retention in continuously operating membrane reactors suffers from the disadvantage that enzymes often do not accept the cofactor derivatives. To overcome this problem Obón et al. proposed a new concept to retain native NADP(H) without chemical modification inside an enzyme membrane reactor by adding charged soluble polymers, such as polyethyleneimine (PEI), which bind the cofactor electrostatically [24]. The concept was successfully tested in the continuous simultaneous synthesis of gluconic acid (**9**) and glutamic acid (**8**) by coupling the NADP^+^-dependent glucose (**7**) oxidation catalyzed by glucose oxidase with the reductive amination of α-ketoglutaric acid (**6**) catalyzed by glutamate dehydrogenase (Scheme 3). When the process was carried out in a continuously operated enzyme membrane reactor loaded with 1 mM PEI, 80% conversion and 7.8 g L^−1^ day^−1^ space-time yield with respect to **9** could be achieved at the retention time of 12 hours.

**Scheme 4:**
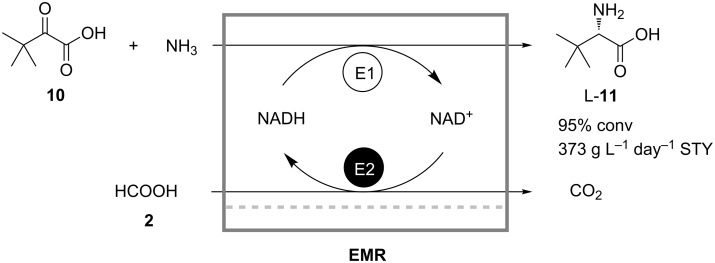
Production of L-*tert*-leucine (**11**) in a continuously operated enzyme membrane reactor equipped with a nanofiltration membrane. E1: Leucine dehydrogenase; E2: Formate dehydrogenase [25].

Seelbach and Kragl showed that the retention of NADH in a continuous synthesis can also be achieved without immobilization or addition of charged polymer, but by using nanofiltration membranes instead of ultrafiltration ones [25]. The reactor for the synthesis of L-*tert*-leucine (**11**) from trimethylpyruvate (**10**) catalyzed by leucine dehydrogenase (Scheme 4) was operated for 10 days with a total turnover number increased by a factor of 3.4 compared to a reactor without cofactor retention. The regeneration of NADH was achieved in a coupled biocatalytic oxidation of formate (**2**).

**Scheme 5:**
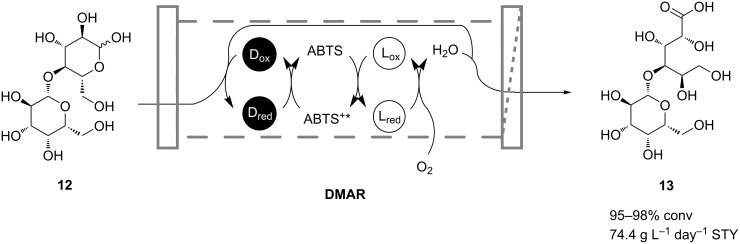
Continuous oxidation of lactose (**12**) to lactobionic acid (**13**) in a dynamic membrane-aerated reactor (DMAR) catalyzed by cellobiose dehydrogenase (D) and laccase (L) [26].

Hecke and coworkers described the production of lactobionic acid (**13**) from lactose (**12**) in a coupled two-enzyme reaction both in discontinuous- and continuous-operation modes (Scheme 5) [26]. Cellobiose dehydrogenase was applied to catalyze the oxidation of **12**, while laccase was used as a regenerating enzyme coupled by the redox mediator 2,2-azino-bis(3-ethylbenzothiazoline-6-sulfonic acid) (ABTS). In continuous-flow experiments, the enzymes were retained by using an ultrafiltration membrane, whereas **12** and also ABTS were fed continuously. Reactions were carried out on a 20 L scale in a dynamic membrane-aeration reactor, which offered the advantage of bubble-free aeration and thus avoiding enzyme deactivation at a gas–liquid interface. The reactor was operated for 3 days and maintained ~80% of the initial enzyme activity. Only microbial contamination prevented a longer operation time of the process as it was carried out under nonsterile conditions. An overall space-time yield of 74.4 g L^−1^ day^−1^ was calculated with conversions in the range of 95–98%.

**Scheme 6:**
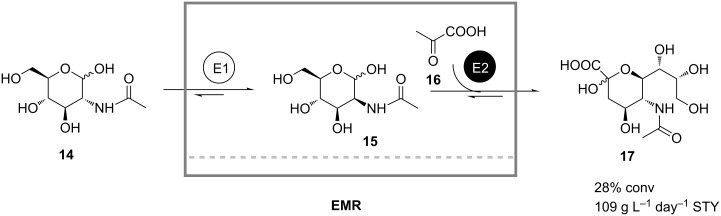
Production of *N*-acetylneuraminic acid (**17**) in a continuously operated enzyme membrane reactor. E1: Epimerase; E2: Aldolase [27].

The continuous enzymatic synthesis of *N*-acetylneuraminic acid (**17**) from *N*-acetylglucosamine (GlcNAc, **14**) in an enzyme membrane reactor employing two enzymes was developed by Kragl and coworkers [27]. In their coupled-reaction system the first enzyme GlcNAc 2-epimerase catalyzed the epimerization of **14** yielding the epimer *N*-acetylmannosamine (**15**), which consequently was condensed by the second enzyme aldolase with pyruvic acid (**16**) to form the product **17** (Scheme 6). By appropriately adjusting the reaction parameters, such as pH, temperature and substrate concentrations, the authors minimized cross-inhibition effects, maintained high enzyme activities and shifted the chemical equilibrium towards the product side. A space-time yield of 109 g L^−1^ day^−1^ was obtained.

**Scheme 7:**
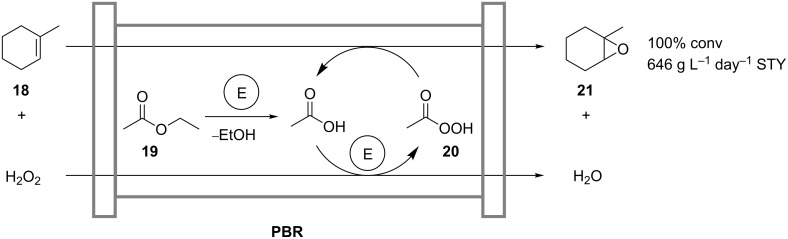
Chemo-enzymatic epoxidation of 1-methylcyclohexene (**18**) in a packed-bed reactor (PBR) containing Novozym 435 (E) [28].

Wiles and coworkers transferred a coupled chemoenzymatic reaction for the oxidation of alkenes from batch operation to an efficient continuous-flow process (Scheme 7) [28]. The process involves the lipase-catalyzed in situ formation of peracetic acid (**20**) from hydrogen peroxide and ethyl acetate (**19**), which oxidizes the model substrate 1-methylcyclohexene (**18**) to form the product 1-methylcyclohexene oxide (**21**). The coupled reaction is carried out in a continuously operated packed-bed microreactor. As compared to the batch-mode experiments, higher concentrations of H_2_O_2_ were applied without detectable catalyst deactivation after 24 hours. At 100% conversion, a space-time yield of 646 g L^−1^ day^−1^ was obtained.

**Scheme 8:**
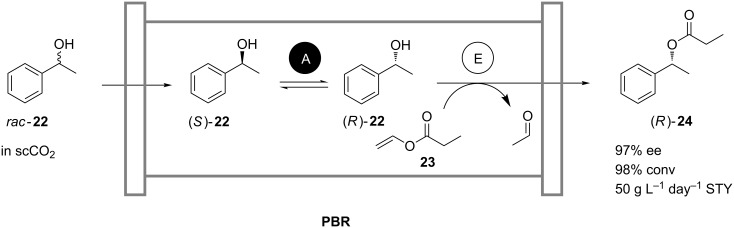
Continuous production of (*R*)-1-phenylethyl propionate (**24**) by dynamic kinetic resolution of (*rac*)-1-phenylethanol (**22**) in supercritical CO_2_ in a packed-bed reactor loaded with acidic zeolite (A) and Novozym 435 (E) [30].

Lozano and coworkers reported on a continuous, chemoenzymatic dynamic kinetic resolution (DKR) process for the production of (*R*)-1-phenylethyl propionate (**24**) from (*rac*)-1-phenylethanol (**22**) and vinyl propionate (**23**) (Scheme 8) [29–31]. In a multiphase packed-bed reactor, commercially available immobilized *Candida antarctica* lipase B (Novozym 435) was used as a heterogeneous catalyst in the kinetic resolution of the alcohol **22** [30]. Racemization of the unreacted (*S*)-**22** was achieved with acidic zeolite catalysts. Both heterogenous catalysts were coated with ionic liquids for improved stability of the lipase in the presence of the acidic chemocatalysts and for reduction of zeolite-catalyzed side reactions [30–31]. The substrates and supercritical CO_2_ were fed continuously, thus omitting the use of organic solvents for product extraction. An enantiomeric excess of 97% was achieved for the product (*R*)-**24** at 98% conversion and a space-time yield of 50 g L^−1^ day^−1^. Operational stability was maintained over a period of 14 days. Previously applied *Candida antarctica* lipase B immobilized on modified C4-silica proved to be unstable upon combination with acidic chemocatalysts. Separation of the acidic chemocatalyst from the lipase by spatial separation in a single column [29] or in consecutive packed-bed reactors [31], however, similarly allowed the process to be efficiently run with high enantiomeric excesses of up to 99%, but with a reduced yield of 60%.

**Scheme 9:**
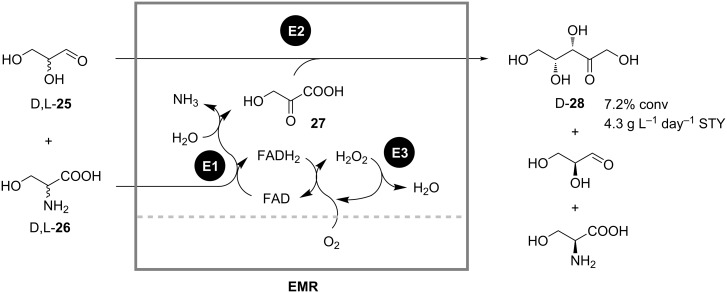
Synthesis of D-xylulose (**28**) from D,L-serine (**26**) and D,L-glyceraldehyde (**25**) in a continuously operated enzyme membrane reactor. E1: D-Amino acid oxidase; E2: Transketolase; E3: Catalase [32].

A reaction sequence for the continuous synthesis of D-xylulose (**28**) from D,L-serine (**26**) and D,L-glyceraldehyde (**25**) involving three enzymes in a single continuously stirred tank reactor (CSTR) was presented by Bongs (Scheme 9) [32]. Here, a D-amino acid oxidase was used to form hydroxypyruvic acid (**27**) from D-**26**, which was subsequently converted to D-**28** by a transketolase-catalyzed reaction with added D,L-**25**. Catalase was used to regenerate the reduced cofactor FADH by oxidation with oxygen. In order to decrease enzyme deactivation caused by shear forces, a bubble-free aeration through a silicon membrane was implemented. A space-time yield of 4.3 g L^−1^ day^−1^ was achieved. However, further optimization of the process was not feasible due to the complexity of the system caused by possible cross-inhibition, deactivation, and reactant instabilities.

All of the single-reactor continuous (chemo)enzymatic processes mentioned, except for the one described by Bongs (Scheme 9), employed a maximum of two different catalysts. To the best of our knowledge, until now there is no established continuous (chemo)enzymatic process catalyzed by four or more isolated catalysts combined in one pot. Therefore, it seems that a simple single-reactor or “in-pot” approach would hardly cope with more complex multicatalyst systems. Even in the three-enzyme process Bongs already encountered problems preventing further optimization of the process. There are three reasons for this: (a) Different enzymes usually work best under different, sometimes even incompatible, reaction conditions, e.g., pH, temperature, ionic strength, or the presence of metal ions; (b) intermediates or mediators of certain steps can inhibit enzymes catalyzing other steps (cross-inhibition); (c) certain enzymes may act on the reactants involved in other steps, and thus catalyze undesirable side reactions. Nevertheless, it may be assumed that in the future continuous multistep (chemo)enzymatic processes involving more than three catalysts will be realized in a single reactor as well. For instance, Liu et al. developed a packed-bed reactor containing seven enzymes co-immobilized through hexahistidine tags on nickel–agarose beads (“super beads”) [33]. These enzymes catalyze a complex network of sequential and coupled reactions, in which galactose, uridine monophosphate (UMP) and inorganic polyphosphate are converted to uridine diphospate galactose (UDP-Gal) in the presence of catalytic amounts of ATP and glucose 1-phosphate. When the reaction mixture containing starting materials and cofactors was circulated for 48 hours through the reactor, 50% of UMP was transformed to UDP-Gal. Although in their work the authors did not establish the continuous production of UDP-Gal, in principle such a reactor could be operated in continuous mode as well, if the immobilized enzymes were more active and stable enough.

### 2. Cascade-reactor processes in vitro

**Scheme 10:**
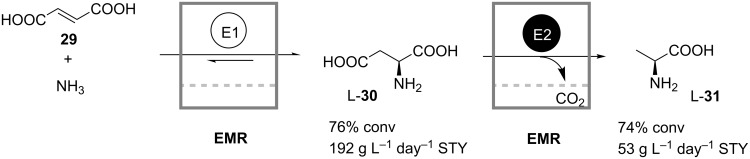
Continuous production of L-alanine (**31**) from fumarate (**29**) in a two-stage enzyme membrane reactor. E1: Aspartase; E2: L-Aspartate-β-decarboxylase [34].

The drawbacks of single-reactor coupled-reaction enzymatic processes arising from incompatibility of individual reaction steps may be overcome in cascade-reactor systems in which the conflicting steps are spatially separated. This idea was recognized and realized already in 1982 by Jandel et al. in their continuous production of L-alanine (**31**) through two consecutive biotransformations conducted in a two-stage EMR cascade [34]. In the first reactor L-aspartic acid (**30**) is formed from fumaric acid (**29**) and ammonia through the action of a soluble aspartase isolated from *E. coli* (Scheme 10)*.* Subsequently, in the second reactor L-**30** is irreversibly decarboxylated by L-aspartate-β-decarboxylase to produce L-**31** and CO_2_. Both enzymes differ significantly in their pH and temperature optima, thus necessitating two separate consecutive reactors. During the operation of the reactor cascade the loss of productivity due to enzyme deactivation in both reactors was compensated by automated enzyme addition. The enzyme consumption required to maintain constant productivity was found to depend on the conversion level. It was also shown that the decarboxylase was deactivated much faster than the aspartase, probably due to the shear forces caused by the formation of CO_2_ bubbles and by recirculating pumps. A space-time yield of approximately 53 g L^−1^ day^−1^ at 56% of overall conversion was achieved.

**Scheme 11:**
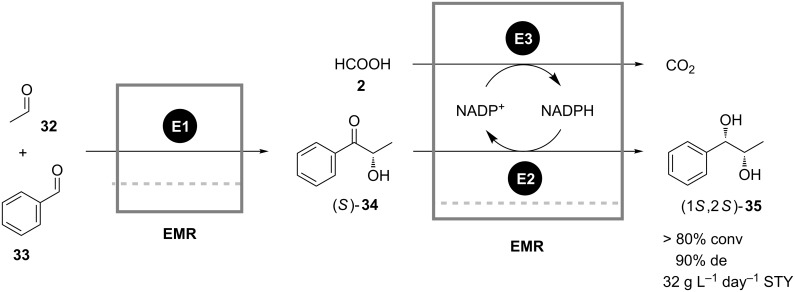
Continuous synthesis of 1-phenyl-(1*S*,2*S*)-propanediol (**35**) in a cascade of two enzyme membrane reactors. E1: Benzoyl formate decarboxylase; E2: Rec*Lb*-alcohol dehydrogenase; E3: Formate dehydrogenase [2].

In the continuous sequential synthesis of *vic*-diols in a multicatalyst system described by Liese [2], the incompatible reaction steps were successfully separated in a cascade of two enzyme membrane reactors as well. The first reactor contained benzoyl formate decarboxylase (BFD) catalyzing the (*S*)-selective aldol condensation of benzaldehyde (**33**) and acetaldehyde (**32**) with formation of (*S*)-2-hydroxy-1-phenyl-propanone (**34)** (Scheme 11). In the second vessel the intermediate (*S*)-**34** was asymmetrically reduced by rec*Lb*-alcohol dehydrogenase (ADH) and the cofactor was regenerated by coupled formate (**2**) oxidation catalyzed by formate dehydrogenase (FDH). Due to the high *K*_M_ value of BFD for **32** an excessive concentration of this compound had to be applied in the first reactor in order to maximize the productivity. Since the aldehyde **32** is also a substrate for the ADH, it was removed inline by stripping with nitrogen in a membrane-based gas–liquid contactor installed before the second reactor. Such an experimental setup enabled continuous synthesis of 1-phenyl-(1*S*,2*S*)-propanediol (**35**) with 90% de and overall space-time yield of 32 g L^−1^ day^−1^.

**Scheme 12:**
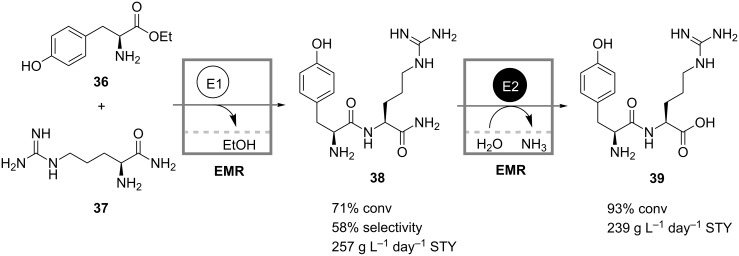
Production of a dipeptide **39** in a cascade of two continuously operated membrane reactors. E1: Carboxypeptidase Y; E2: Peptide amidase [35].

Similarly, Schwarz and Wandrey developed a cascade of two membrane reactors for the production of dipeptides [35]. In the first reactor carboxypeptidase Y (CPD-Y) catalyzes the condensation between the ester **36** and the amide **37** of two amino acids (Scheme 12). Deamidation of the resulting dipeptide-amide **38** proceeds in the second reactor loaded with a selective peptide-amidase that does not react on dipeptide bonds. In a slow side reaction, however, CPD-Y hydrolyzes the final deamidated dipeptide **39**, whereas it does not act on the intermediate **38**. Separation of the two reactions was thus preferred in order to maximize yields.

**Scheme 13:**
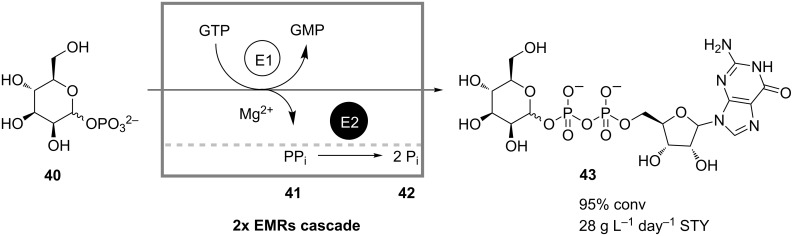
Continuous production of GDP-mannose (**43**) from mannose 1-phosphate (**40**) in a cascade of two enzyme membrane reactors. E1: GDP-mannose pyrophosphorylase; E2: Pyrophosphatase [36].

The cascade-reactor approach may be useful not only in situations where incompatible reaction steps need to be spatially separated, but also in the case of coupled-reaction processes that, from a reaction-engineering point of view, should be conducted in CSTR series. For instance, when developing a continuous enzymatic production of GDP-mannose **43** from mannose 1-phosphate (**40**) using GDP-mannose pyrophosphorylase (Scheme 13), Fey and co-workers encountered a two-fold product inhibition of the enzyme by both products **43** and pyrophosphate **41**, which deteriorated production performance [36]. Inhibition by pyrophosphate was successfully circumvented by applying the second enzyme pyrophosphatase, which catalyzed the hydrolysis of **41** to the noninhibiting inorganic phosphate **42**. Strong product inhibition by the target product **43** was avoided by means of reaction engineering: The process was conducted in a cascade of two CSTRs, whose kinetic behavior approximates an optimum for this reaction system in a plug-flow reactor. In this way the authors were able to reach 95% conversion with a space-time yield of 28 g L^−1^ day^−1^ and an enzyme consumption of 0.9 U g^−1^. The reactor cascade was stably operated for a period of 50 hours.

**Scheme 14:**
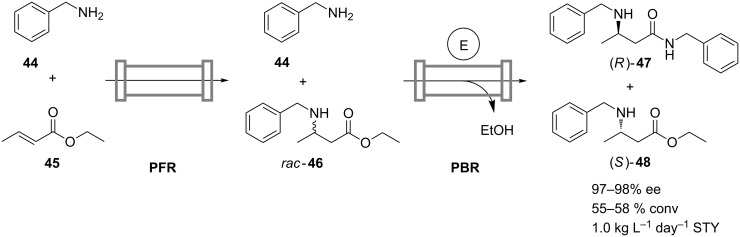
Continuous solvent-free chemo-enzymatic synthesis of ethyl (*S*)-3-(benzylamino)butanoate (**48**) in a sequence of a plug-flow reactor (PFR) and a packed-bed reactor containing Novozym 435 (E) [38].

The potential of the cascade-reactor approach is also appreciated in continuous chemo-enzymatic sequences, where overcoming the intrinsic incompatibility of chemical and enzymatical reaction steps is a common problem. Based on the one-pot batch process described by Weiß and Gröger [37], Strompen et al. developed a continuous chemo-enzymatic synthesis of ethyl (*S*)-3-(benzylamino)butanoate (**48**), which is a precursor to (*S*)-3-aminobutanoic acid. In contrast to the multistep enzymatic processes previously mentioned here, this transformation is carried out in a nonaqueous and solvent-free environment [38]. In the first noncatalyzed thermal aza-Michael addition performed at 80 °C, the racemic ester *rac*-**46** is formed from cheap starting materials benzylamine (**44**) and *trans*-ethylcrotonate (**45**) (Scheme 14). Subsequently, Novozym 435 is applied for the kinetic resolution of *rac*-**46** through aminolysis with the excess of the amine **44**. The resolution is conducted at 60 °C and yields the corresponding (*R*)-amide **47** and the remaining essentially enantiopure ester (*S*)-**48** as the products. Due to the advantage provided by entirely omitting the use of solvents, a high space-time yield of 1 kg L^−1^ day^−1^ was achieved. Apart from the difference in operation temperatures, separation of the two steps of this reaction sequence into individual reactors was also necessary as Novozym 435 is also able to catalyze the unwanted aminolysis of **45**.

**Scheme 15:**
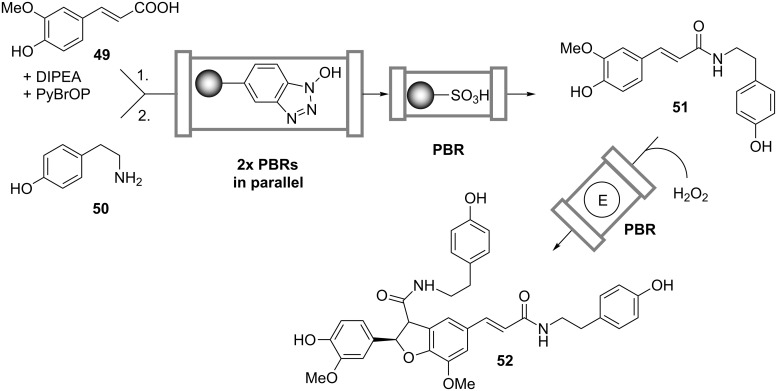
Continuous chemo-enzymatic synthesis of grossamide (**52**) in a cascade of packed-bed reactors. E: Peroxidase [39].

Baxendale et al. [39] established a continuous synthesis of the neolignan natural product grossamide (**52**) by a two-step chemo-enzymatic reaction cascade starting from ferulic acid (**49**) and tyramine (**50**) (Scheme 15). The synthesis was performed in a fully automated flow reactor consisting of three types of prepacked columns. The first two columns connected in parallel were filled with polymer-supported hydroxybenzotriazole, and during the process they worked either in loading or in elution mode. In the loading mode the columns were flushed with a solution of **49**, bromo-tris-pyrrolidino-phosphonium hexafluorophosphate (PyBrOP) and diisopropylethylamine (DIPEA), while in the elution mode a solution of **50** was pumped through. In this manner the acid **49** was first activated in the form of an ester, which then reacted with the amine **50** to yield the intermediate amide **51**. The unreacted **50** was removed from the reaction stream when passing through the scavenger column containing a sulfonic acid resin. The H_2_O_2_-mediated oxidative dimerization and intramolecular cyclization of **51** to the product **52** was catalyzed by an immobilized peroxidase enzyme packed into the last column. The authors validated the design of the flow reactor by synthesizing gram quantities of the compound and suggested that such a setup can be used for the continuous multistep synthesis of a much wider range of chemical substances.

**Scheme 16:**
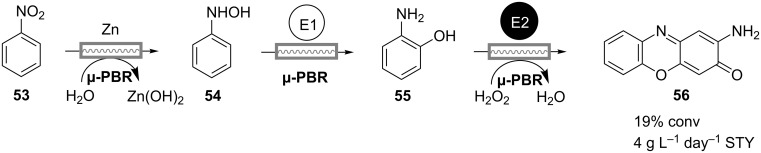
Chemo-enzymatic synthesis of 2-aminophenoxazin-3-one (**56**) in a cascade of continuously operating packed-bed microreactors. E1: Hydroxyaminobenzene mutase; E2: Soybean peroxidase [40].

A sequential chemo-enzymatic reaction in continuous flow was also developed by Luckarift and coworkers [40]. Their three-step process comprises reduction of nitrobenzene (**53**) by zinc to hydroxyaminobenzene (HAB, **54**), which in the second HAB-mutase-catalyzed step is rearranged intramolecularly to form 2-aminophenol (**55**) (Scheme 16). The final transformation involves the oxidation of **55** to 2-aminophenoxazin-3-one (**56)** by H_2_O_2_, catalyzed by soybean peroxidase. All reactions are carried out in an aqueous microfluidic system consisting of a cascade of three microreactors separately loaded with zinc powder and immobilized enzymes. The observed low conversion of 19% and space-time yield of 4 g L^−1^ day^−1^ imply the need for optimization, but demonstrate the feasibility and applicability of this process for the screening of nitroarene conversions.

**Scheme 17:**
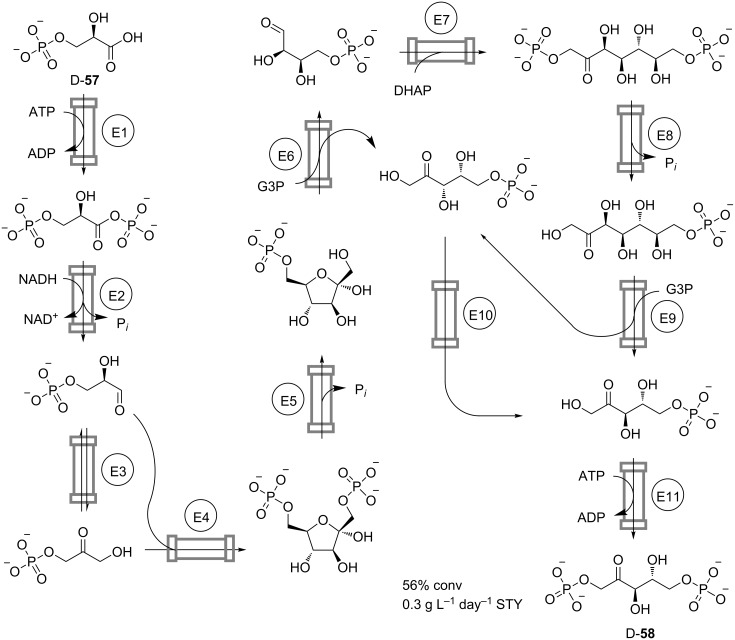
Continuous conversion of 3-phospho-D-glycerate (**57**) into D-ribulose 1,5-bisphosphate (**58**) in a cascade of packed-bed reactors. E1: Phosphoglycerate kinase; E2: Glycerate phosphate dehydrogenase; E3: Triose phosphate isomerase; E4: Aldolase/FBP; E5: Phosphatase/FBPase; E6: Transketolase; E7: Aldolase/SuBP; E8: Phosphatase/SBPase; E9: Transketolase; E10: Epimerase; E11: Phosphoribulokinase [41].

The cascade-reactor approach is also powerful for the multistep enzymatic processes involving more than three reaction steps. An illustrative example is the continuous conversion of D-3-phosphoglycerate (**57**) into D-ribulose 1,5-bisphosphate (**58**) in a series of eleven packed-bed reactors containing immobilized enzymes (Scheme 17) [41]. The aim of the process is to continuously regenerate D-**58**, which is used as an acceptor for the biocatalytic fixation of CO_2_ resulting in the formation of two molecules of D-**57**. The authors achieved 56% overall conversion at 0.3 g L^−1^ day^−1^ space-time yield and, thus, demonstrated the feasibility of carrying out such complex sequences in continuous flow. However, the observed overall conversion was significantly lower than the 90% predicted from enzyme performances in individual reaction steps. This discrepancy was explained by possible modulation of the kinetic properties of the enzymes by the residual intermediates, cofactors, and by-products from previous incomplete enzymatic conversions.

Although the cascade-reactor approach to continuous coupled (chemo)enzymatic reactions allows one in many cases to overcome the challenge of incompatible reaction steps, its practical usefulness is limited to the systems in which every reaction step itself proceeds efficiently. For example, if the conversion after each step in the reaction cascade is 80%, which is usually an excellent value for most single-reaction organic syntheses, after five consecutive steps the overall conversion of such a process will be dramatically decreased to only 33%. Moreover, the presence of the remaining nonconverted intermediates increases the chance of possible cross-inhibition and complicates the isolation of the target compound.

**Scheme 18:**
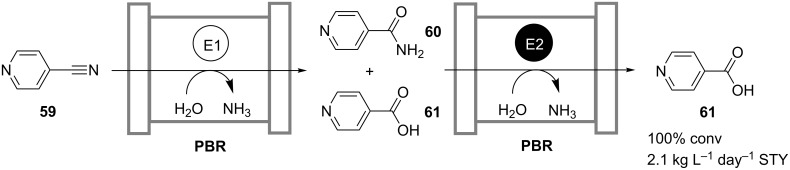
Continuous hydrolysis of 4-cyanopyridine (**59**) to isonicotinic acid (**61**) in a cascade of two packed-bed reactors. E1: Nitrilase; E2: Amidase [42].

Certainly, one way to solve this problem is to find better catalysts for problematic steps or, by means of catalyst engineering, to improve the existing ones. Another plausible way to enhance the overall coupled-reaction process performance is “to polish” inefficient reaction steps with the aid of hybrid-reactor technology, which combines reaction and downstream processing steps in situ for the removal of undesired by-products. The work of Martinkova and coworkers, who observed the formation of isonicotinamide (**60**) as a by-product in the nitrilase-catalyzed conversion of 4-cyanopyridine (**59**) to isonicotinic acid (**61**), serves as an illustration of this method [42]. There, a cascade of two packed-bed reactors was set up with the second reactor loaded with an immobilized amidase, which catalyzed the hydrolysis of the residual amide **60** (Scheme 18). Both reactions were carried out in an aqueous system, while care was taken to find suitable buffer salts acceptable for both enzymes. A space-time yield of 2.1 kg L^−1^ day^−1^ was obtained at full conversion and 99.8% purity of the desired product. The same reaction sequence, but catalyzed by enzymes of a different origin, was realized by Malandra et al. in two enzyme membrane reactors operated in series [43].

### 3. Whole-cell processes in vivo

If living cells are the most efficient chemical factories known up to now, then why not let them do multistep organic synthesis for our benefit? This fact was already recognized before 6000 BC when the ancient Sumerians and Babylonians practiced the brewing of beer and winemaking [6]. Indeed, in vivo fermentation of sugars to ethanol by yeast is a complex process involving at least 12 enzymes and 2 cofactors, NAD^+^ and ADP. Nowadays microbial biotransformations have become an indispensable part of industrial biotechnology, and are used in the production of a broad range of bulk and fine chemicals [6]. When a microbial multistep biotransformation is carried out in continuous flow it might formally also be considered as a fourth-generation enzymatic process according to the above-mentioned classification. However, in contrast to the in vitro multistep transformations already discussed, the whole-cell processes proceed in vivo, and, therefore, they are treated separately herein.

There are many continuous microbial biotransformations/fermentations described in the literature, for example fermentative production of ethanol, butanol, lactic acid, acetic acid/vinegar, succinic acid and fumaric acid in continuously operated biofilm reactors containing thick layers of microbial cells [44]. In this section only a few representative examples are reviewed, which illustrate the potential and the drawbacks of the whole-cell approach to continuous enzymatic coupled-reaction processes.

**Scheme 19:**
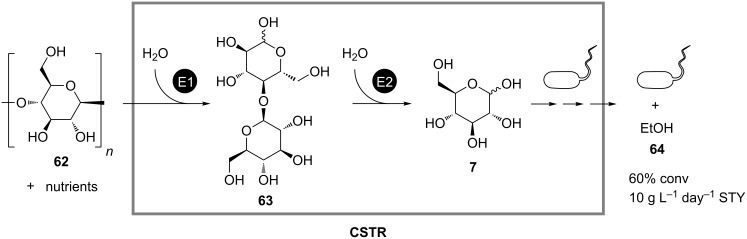
Continuous fermentative production of ethanol (**64**) from hardwood lignocellulose (**62**) in a stirred-tank reactor (CSTR) containing yeast cells, cellulase (E1) and β-glucosidase (E2) [45].

One of these examples is the continuous fermentative production of ethanol (**64**) developed by South et al. It is based on degradation of hardwood lignocellulose (**62**) by application of both free enzymes and whole cells as biocatalysts (Scheme 19) [45]. At the beginning the substrate **62** is pretreated with diluted acid in order to allow continuous feeding of the substrate suspension to a fermenter and to enhance conversion rates. In the fermenter the pretreated **62** is first hydrolyzed to cellobiose (**63**) by a cellulase from *Trichoderma reesi*. Then the second enzyme β-glucosidase breaks **63** down to form glucose (**7**), which is finally fermented by *Saccharomyces cerevisiae* to yield **64**. The side products formed during the acidic pretreatment of **62** cause only minor inhibition effects on the progress of the enzymatic saccharification and fermentation. A 60% conversion was obtained after a residence time of 2 days and with a space-time yield of 10 g L^−1^ day^−1^. This process also demonstrates the applicability of continuous microbial biotransformations in biorefinery – the technology, which is aimed at generating energy from renewable resources and which is actively propagated presently in view of the forthcoming global energy crisis.

**Scheme 20:**
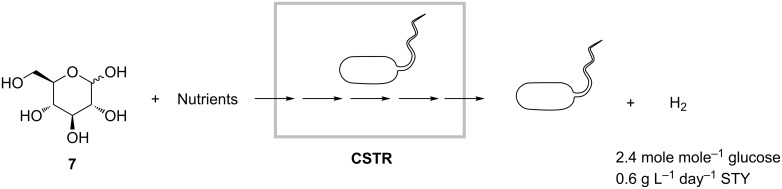
Production of hydrogen by anaerobic fermentation of glucose (**7**) using *Clostridium acetobutylicum* cells in a continuously operating stirred-tank reactor [46].

Another example of a biorefinery process is the production of hydrogen, i.e., the most environmentally friendly fuel. Oh and coworkers realized a continuous anaerobic fermentation of *Clostridium acetobutylicum* strains, which convert glucose (**7**) to hydrogen (Scheme 20) [46]. The reactor was initially operated in batch mode until the redox potential went below −200 mV. Afterwards, the reactor was continuously fed with a solution of **7**. During one week of continuous operation at 30 °C and retention time of 10 hours, approximately 2.4 mole of H_2_ per mole of glucose were produced with a space-time yield of 0.6 g L^−1^ day^−1^. Fermentation by-products comprised mainly butyrate and acetate as well as low amounts of ethanol and butanol.

Microbial biotransformations were also successfully exploited in the continuous multistep enzymatic synthesis of fine chemicals, where cofactor regeneration is required. In such cases the whole-cell in vivo approach is advantageous over in vitro approaches, because it is not necessary to use immobilization or nanofiltration membranes for the retention of small cofactor molecules when whole cells are applied as biocatalysts. Instead, the cofactors are kept in the cytoplasm by the cell membrane, which functions as a natural and efficient diffusion barrier. Moreover, the enzymes are usually more stable in their native environment inside a living cell than they are in buffer solutions or in immobilized forms.

**Scheme 21:**
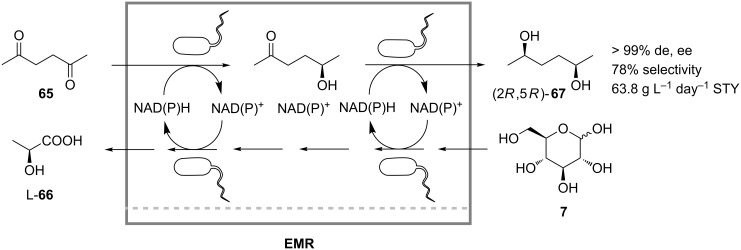
Continuous production of (2*R*,5*R*)-hexanediol (**67**) in an enzyme membrane reactor containing whole cells of *Lactobacillus kefir* [47].

*Lactobacillus kefir* whole cells were used by Haberland et al. for the continuous production of (2*R*,5*R*)-hexanediol (**67**) from 2,5-hexanedione (**65**) by in vivo reaction sequence in an ultrafiltration membrane reactor (Scheme 21) [47]. Native *L. kefir* alcohol dehydrogenases catalyzed the enantioselective and diastereoselective reduction of the ketone **65** while the reduction equivalents were supplied by the cosubstrate glucose. During the process the cells produced one mole of lactic acid (**66**) per mole of glucose (**7**) by fermentation. Therefore, NaOH was constantly added to the reactor to maintain the pH. The production facility also included an in-line product-separation unit consisting of a continuously operated counter-current extraction unit, and an online distillation unit for recycling of the organic solvent. The reaction product (2*R*,5*R*)-**67** was obtained at a space time yield of 63.8 g L^−1^ day^−1^ and a selectivity of 78%.

The biocatalytic reduction of **65** to (2*R*,5*R*)-**67** was also accomplished by Schroer and Luetz in a continuously operated double-membrane reactor loaded with recombinant *E. coli* cells overexpressing an alcohol dehydrogenase from *Lactobacillus brevis* [48]. In contrast to the work of Haberland [47], the consumed cofactor NADPH was regenerated by coupling the reduction of the ketone **65** with the oxidation of 2-propanol to acetone. The encountered thermodynamic limitation of the reaction system was overcome by continuous product removal of acetone by pervaporation (vaporization by permeation) through a polymethoxysiloxane membrane. To reach the highest space-time yields of >170 g L^−1^ day^−1^, the reactor was constantly fed with a 10 mM solution of NADP; although without addition of the cofactor the reactor also revealed high productivity and operational stability.

**Scheme 22:**
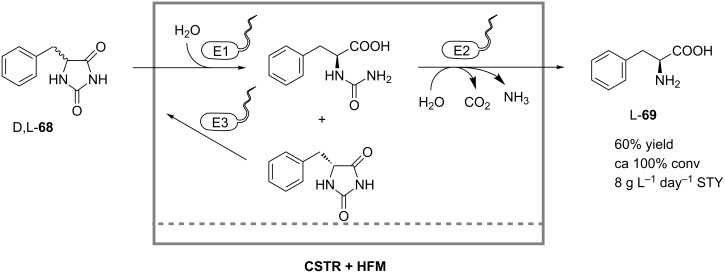
Synthesis of L-phenylalanine (**69**) in a continuously stirred tank reactor equipped with a hollow-fiber module (HFM) for retention of *Arthrobacter aurescens* cells. E1: Hydantoine racemase; E2: L-Hydantoinase; E3: L-*N*-Carbamoylase [49].

The multistep microbial biotransformations are also feasible for the continuous processes that do not require cofactor regeneration. Nöthe and coworkers developed a whole-cell membrane reactor operated in CSTR mode, with continuous product removal by ultrafiltration, for the microbial conversion of D,L-5-monosubstituted hydantoines [49]. Resting whole cells of *Arthrobacter aurescens* containing a hydantoine racemase, an L-hydantoinase and an L-*N*-carbamoylase were used to catalyze the synthesis of L-phenylalanine (**69**) from the model substrate D,L-benzylhydantoine (**68**) (Scheme 22) in 60% yield at almost 100% substrate conversion and a space-time yield of 8 g L^−1^ day^−1^. Shear forces introduced by pumps were responsible for irreversibly damaging the cell walls, thus causing an increased enzyme deactivation rate, after an initial gain in activity from the improved permeability of substrates through the cell wall. Additionally, membrane fouling was identified as a problem for long-term operation. After several rounds of optimization, this multistep L-hydantoinase process was commercialized by DSM for industrial production of natural and non-natural L-amino acids [50].

**Scheme 23:**
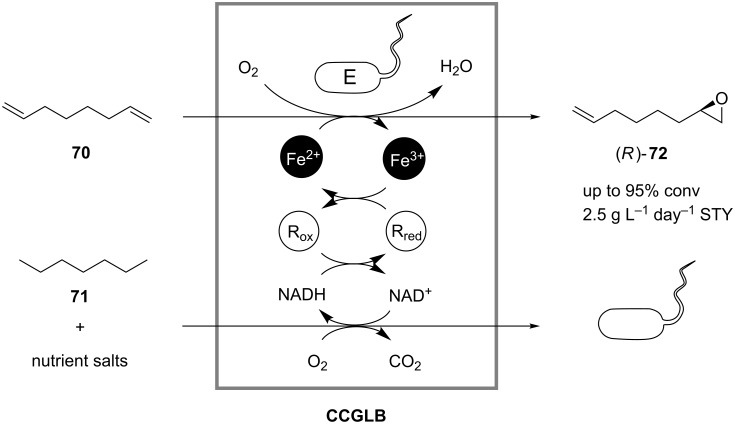
Continuous epoxidation of 1,7-octadiene (**70**) to (*R*)-7-epoxyoctene (**72**) by a strain of *Pseudomonas oleovorans* in a closed-gas-loop bioreactor (CCGLB). R: Reductase; Fe: Rubredoxin [51].

A serious disadvantage of the whole-cell approach for continuous coupled-reaction processes is the sensitivity of living cells to high concentrations of organic substances, which are usually toxic. To overcome this challenge, Steinig and coworkers developed a special closed-gas-loop bioreactor and used it for the continuous epoxidation of 1,7-octadiene (**70**) to (*R*)-7-epoxyoctene (**72**) by a strain of *Pseudomonas oleovorans* growing on heptane (**71**) (Scheme 23) [51]. In a continuous operation, with regard to the aqueous phase, substrates for both growth and biotransformation were supplied in the gas phase from a reservoir and were dissolved in the liquid phase containing the whole cells and other nutrients required for the cell growth. The product was again removed in the gas phase and collected in a saturator/absorber module. By supplying and removing substrates and products by transfer to the gas phase, problems related to cell toxicity in the presence of organic phases or emulsified product streams were avoided. A space-time yield of approximately 2.5 g L^−1^ day^−1^ was achieved.

**Scheme 24:**
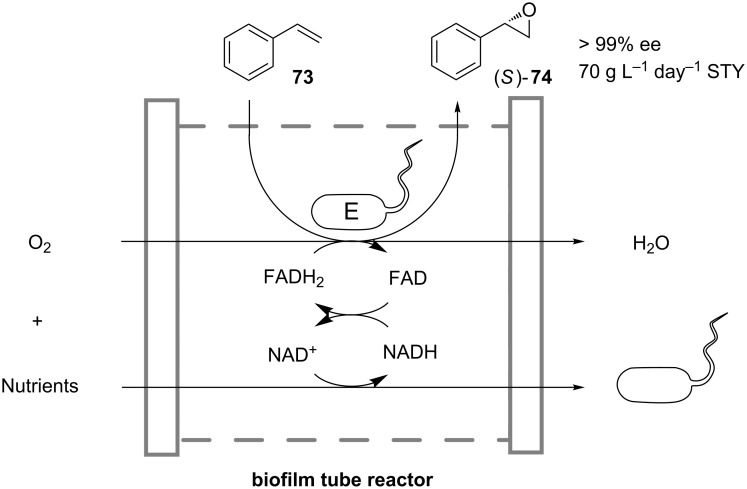
Oxidation of styrene (**73**) to (*S*)-styrene oxide (**74**) in a continuously operated biofilm tube reactor containing cells of *Pseudomonas sp.* [53].

Another method to surmount substrate toxicity was used by Gross et al. in the oxidation of styrene (**73**) to (*S*)-styrene oxide (**74**) in a biofilm membrane reactor (Scheme 24) [52–53]. Cells of *Pseudomonas sp.* were grown in a biofilm attached to the inner surface of a silicon tube, through which a nutrient solution was constantly pumped. In a specially designed hermetic reaction compartment the tube was partially submerged into liquid **73**, which slowly diffused through the tube wall to the biofilm. Inside the tube **73** was enantioselectively oxidized to (*S*)-**74**, which in turn diffused back into the reaction compartment. The cofactor FADH_2_ required for the oxidation was regenerated during metabolic activity of the cells. Due to diffusion limitations the substrate and the product concentrations in the biofilm were lower than their toxic levels, which allowed for continuous operation of the reactor for a period of more than 50 days, reaching maximally a space-time yield of 70 g L^−1^ day^−1^.

**Scheme 25:**
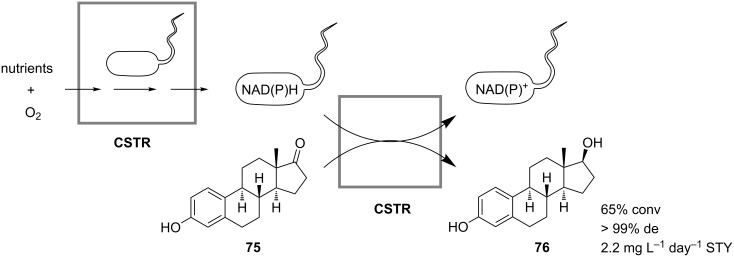
Reduction of estrone (**75**) to β-estradiol (**76**) by *Saccharomyces cerevisiae* in a cascade of two stirred-tank reactors [54].

Cheng and Tsai encountered the problem of substrate inhibition and low product yields during the reduction of estrone (**75**) to β-estradiol (**76**) in batch cell cultures of *Saccharomyces cerevisiae* expressing a native enzyme 17β-hydroxysteroid dehydrogenase. Therefore, in order to increase the productivity of cells, the authors proposed to perform the process continuously in a cascade of two stirred-tank reactors [54]. In the first reactor a continuous cell culture of the yeast was grown aerobically and continuously fed to the second reactor, in which reduction of **75** took place (Scheme 25). The constant flux of fresh active cells from the first reactor maintained the productivity of the second reactor at a constant level, which otherwise would drop down due to substrate-promoted cell inactivation and consequent cell wash-out. In the continuous process the productivity of yeast cells was improved compared to batch operation, giving a yield of 64.8% of the steroid **76**, and the overall product recovery was increased by a factor of about 4.3. A space-time yield of approximately 2.2 mg L^−1^ day^−1^ was achieved after three days of operation.

## Conclusion

The examples of continuous coupled (chemo)enzymatic reactions reviewed herein demonstrate that such biotransformations can be considered as workable and competitive synthetic routes in the organic synthesis of fine and bulk chemicals. However, despite its obvious potential, this technology is presently in its infancy and the number of successful practical implementations is rather small; this number will certainly increase significantly in the future. There is no universal approach or recipe for how to build up a novel, efficient fourth-generation enzymatic process, but there are three different approaches, namely the single-reactor, cascade-reactor and whole-cell approach, the application of which in a particular coupled-reaction enzymatic system can be advantageous or disadvantageous (Table 1) depending on the process criteria under consideration. It is anticipated that in the future all three approaches will be further optimized in order to minimize their drawbacks and to maximize their positive sides. In this respect the exploitation of other biological principles could be of advantage and could lead to the next generation of enzymatic processes.

**Table 1:** Advantages and disadvantages of the three approaches to continuous coupled-reaction (chemo)enzymatic processes.^a^

Criterion	in vitro single reactor	in vitro cascade reactor	in vivo whole cell

Separation of incompatible steps	−	+	−

Incorporation of chemical steps	0	+	−

Optimization by reaction engineering	0	+	−

Modularization/incorporation of downstream processing units	−	+	−

Enzyme preparation/costs	−	−	+

Cofactor regeneration	0	−	+

Atomic efficiency	0	0	−

^a^(+) advantage; (−) disadvantage; (0) no general comment possible, dependent on respective reaction system.

For instance, another biological principle states that the cell metabolism is compartmentalized, i.e., the interior of a living cell consists of compartments clustering the enzymes of a given metabolic pathway. Moreover, the clustered enzymes usually form supramolecular complexes enabling so-called “substrate channeling”, where intermediates of sequential metabolic steps are not released into the cytoplasm, but instead are “channeled” from one enzyme to another. This principle has been recently utilized in applied biocatalysis for the development of novel types of catalysts for multistep reaction cascades, i.e., metabolon catalysts [55], self-assembled fusion protein complexes [56] and polymerosomes [57]. It is very probable that application of these novel catalysts in continuous “in-pot” coupled-reaction processes will facilitate the separation of incompatible reaction steps, and thus can help to overcome the main disadvantage of a single-reactor approach.

A common drawback for both in vitro approaches is the stability of the enzyme preparations used, which is usually too low to make the long-term operation of the processes economically attractive. Furthermore, the activity and selectivity of enzymes in the non-natural environment is often deteriorated, which has a negative impact on the overall process performance. The enhancement of enzyme properties, or “fitting” them to a particular operation window by means of directed evolution or protein design [58], is a good way to make continuous coupled-reaction (chemo)enzymatic processes more appealing for practical purposes. And proceeding in this way would represent the realization of one more biological principle, namely that cell metabolism is steadily enhanced or adapted to the changing environment by natural evolution.

The main problem associated with the in vivo whole-cell approach is the complexity of metabolic networks, which is responsible for the comparably low yields and atomic efficiency of the multistep synthesis of target compounds, due to the dissipation of intermediates in numerous side reactions. The amendment of continuous in vivo coupled-reaction processes by metabolic engineering [59] is a promising technology, which brings into play another crucial biological principle, manifesting a control of the metabolic activity of a living cell through its genetic composition. The related and more advanced technology, which in the foreseeable future will revolutionize the field of whole-cell biotransformations, is that of genome engineering as pioneered by Craig Venter [60]. This technology is believed to be the main tool of synthetic biology, envisioning the creation of artificial cells with synthetic metabolic networks programmed to do a specific task, such as the treatment of diseases, the degradation of pollutants, or the synthesis of biofuels, and bulk and fine chemicals. Although there is still a long way to go to establish multistep continuous biotransformations catalyzed by artificial cells, there is no doubt, that when this goal is reached, chemists will be proud to say that they are on their way to being able to compete with nature in the virtuosity of organic synthesis.
